# Prevalence of occult hepatitis B infection and hepatitis B genotype characterization among blood donors in Jember Regency, East Java, Indonesia

**DOI:** 10.1186/s12879-025-11864-9

**Published:** 2025-10-31

**Authors:** Valentinus Dave Sugiharto, Nourhane Hafza, Noer Sidqi Muhammadiy, Rini Riyanti, Le Chi Cao, Dao Thi Huyen, Truong Nhat My, Tran Thi Thanh Huyen, Le Huu Song, Kartika Senjarini, Thirumalaisamy P. Velavan

**Affiliations:** 1 Faculty of Medicine, University of Jember, Jember, 68121 Indonesia; 2 Institute of Tropical Medicine, University of Tübingen, Tübingen, 72074 Germany; 3 Faculty of Pharmacy, University of Jember, Jember, 68121 Indonesia; 4 Department of Parasitology, Hue University of Medicine and Pharmacy, Hue University, Hue, 49000 Vietnam; 5 Vietnamese German Center for Medical Research, VG-CARE, Hanoi, 10000 Vietnam; 6 108 Military Central Hospital, Hanoi, 10000 Vietnam; 7 Department of Biology, Faculty of Mathematics and Natural Sciences, University of Jember, Jember, 68121 Indonesia; 8 Faculty of Medicine, Duy Tan University, Da Nang, 55000 Vietnam

**Keywords:** Hepatitis b, Occult hepatitis b, Blood donors, Indonesia, Nucleic acid testing

## Abstract

**Background:**

Despite extensive HBV vaccination efforts in Indonesia, the prevalence of occult hepatitis B virus infection (OBI) remains a concern, particularly among blood donors. This study investigated the prevalence of OBI, the distribution of circulating HBV genotypes, seropositivity for HBV-specific antibodies, and the potential influence of mutational characteristics on HBsAg secretion among blood donors in Jember, Indonesia.

**Methods:**

A total of 330 HBsAg-negative blood donor samples from the Indonesian Red Cross in Jember were analyzed for HBV serological markers. Qualitative nested PCR and quantitative real-time PCR were used to detect HBV viral DNA and to measure viral load, respectively. HBV genotyping in positive samples was performed using Sanger sequencing of the S gene fragment.

**Results:**

The analysis revealed an OBI prevalence of 3%. Serological testing indicated that 27% of participants were positive for anti-HBs antibodies, 18% for anti-HBc, and 6% for anti-HBc alone. All HBV DNA-positive cases belonged to genotype B, primarily sub genotypes B3 and B4. Phylogenetic analysis revealed multiple mutations in the HBV surface (S) gene and the reverse transcriptase (RT) domain of the polymerase (Pol) protein, including immune escape mutations, suggesting potential risks for HBV transmission and reactivation.

**Conclusions:**

These findings emphasize the need for enhanced screening, including NAT alongside serological testing, to mitigate OBI risks in blood donors and improve transfusion safety and public health policies in HBV-endemic regions of Indonesia.

**Supplementary Information:**

The online version contains supplementary material available at 10.1186/s12879-025-11864-9.

## Introduction

Infectious diseases remain a significant global public health challenge, with viral hepatitis being a major contributor to morbidity and mortality. Chronic hepatitis B virus (HBV) infection is a significant global health challenge, affecting approximately 254 million people worldwide. Indonesia ranks among the top ten countries contributing to nearly two-thirds of these cases [1]. Although chronic HBV infection is often asymptomatic, it can lead to severe liver complications such as cirrhosis and hepatocellular carcinoma, with rising cirrhosis rates observed in affected populations [2].

Occult hepatitis B infection (OBI) is characterized by the presence of HBV covalently closed circular DNA in HBsAg-negative individuals, typically with low viral loads below 200 IU/mL. Despite being often asymptomatic, OBI remains transmissible and can be reactivated, posing risks for high-risk groups such as immunocompromised individuals and patients with chronic liver disease or those who have received blood transfusions [3, 4]. HBV is transmitted through perinatal, blood, and sexual routes, underscoring the importance of immunization and early detection for effective prevention [5]. Indonesia national HBV vaccination program, established in 1997, achieved 85% coverage by 2021 [5, 6]. While rapid diagnostic tests (RDTs) offer convenient screening for hepatitis B, their low sensitivity and inability to detect emerging HBV variants can hinder accurate diagnosis, particularly for OBI [3].

OBI can be classified into seropositive (anti-HBc and/or anti-HBs positive) and seronegative (anti-HBc and anti-HBs negative) groups. Anti-HBc positivity indicates a past or ongoing HBV infection, whereas vaccinated individuals develop anti-HBs without anti-HBc. OBI cases are often anti-HBc positive and are confirmed through nucleic acid testing (NAT) on serum samples. While anti-HBs antibodies may wane over time in vaccinated individuals, OBI cases exhibit variable serological profiles, with some testing positive for anti-HBs and/or anti-HBc, while others remain seronegative. Notably, approximately 20% of OBI cases test negative for all HBV serological markers, making detection particularly challenging. Additionally, these cases may be missed in qualitative assays due to low viremia, further complicating diagnosis [7]. Certain risk factors, such as immunosuppression, co-infections (e.g., HCV or HIV), and host immune responses, can further influence seronegative OBI. These factors may impair antibody production or accelerate antibody loss, reducing detectable serological markers while HBV DNA persists [8, 9].

Across Asia, the prevalence of occult hepatitis B infection varies widely, ranging from 0.013% in China to 10.9% in Laos, with Vietnam reporting a rate of 0.3% [10, 11]. In Indonesia, the overall HBsAg prevalence is 7.1%, indicating a moderate to high HBV infection rate, with genotypes B (66%) and C (26%) are the most common [12, 13]. Indonesia comprises 38 provinces, further divided into regencies. Reported OBI prevalence rates among blood donors range from 8% in Central Java and North Sumatra [6] to 13% in North Maluku [14]. Among healthy students in Central Kalimantan, the prevalence is 7% [15]. Notably, the highest reported OBI was observed in the transgender population in Surabaya, at 21% [16].

Jember, a regency in East Java province with 2.6 million residents, currently lacks specific data on HBV prevalence and infection trends, leaving the prevalence of OBI in Jember and its surrounding areas largely unexplored. This study investigates the prevalence of OBI, defined by the presence of hepatitis B viral DNA in serum, along with associated viral factors and circulating HBV genotypes among blood donors from Jember who tested negative for HBsAg.

## Materials and methods

### Study population

This cross-sectional study was conducted at the Department of Clinical Pathology, University of Jember. A total of 330 HBsAg-negative serum samples were collected from blood donors at the Indonesian Red Cross (PMI; Palang Merah Indonesia) in the Jember region for four consecutive days in March 2024. The eligibility criteria for blood donation in Indonesia include being in good physical and mental health, aged between 17 and 60 years, with a minimum weight of 45 kg. Donors must have blood pressure within the range of 90–160 mmHg (systolic) and 60–100 mmHg (diastolic), and a hemoglobin level between 12.5 and 17 g/dL. A minimum interval of two months between donations is required, and donors must be willing to donate voluntarily. These criteria were applied to all participants in our study. All donors provided written informed consent and demographic data were collected. All donor samples were screened using the chemiluminescent immunoassay (CLIA) and were determined negative for HBsAg. Additionally, all samples were tested negative for anti-HCV, anti-HIV, and syphilis using rapid diagnostic tests according to the PMI Jember standard operating procedures.

### Screening of HBV serological markers

All samples were tested for HBV serological markers including anti-HBs (Monolisa™ anti-HBs PLUS, BIO-RAD, Hercules, CA, USA) and anti-HBc (Monolisa™ total anti-HBc PLUS, BIO-RAD, Hercules, CA, USA) using ELISA procedures. Absorbance values were measured using a CLARIOstar microplate reader (BMG Labtech, Ortenberg, Germany). Anti-HBs positivity was determined quantitatively, defined as a titer value greater than 10 mIU/mL. Anti-HBc was assessed qualitatively, with positivity defined as any value above the manufacturer’s cut-off. Sensitivity and specificity were greater than 99% for all assays.

### Nucleic acid isolation

Serum samples were subjected to nucleic acid isolation using the QIAmp DNA Mini Kit (Qiagen GmbH, Hilden, Germany) following manufacturer’s instructions. Subsequently, the quality and quantity of the DNA were assessed using NanoDrop™ (Thermo Fisher Scientific, Waltham, MA, USA), and subsequently‚ stored at -80 °C until further use.

### Qualitative nested PCR and Sanger sequencing

HBV DNA in serum samples was investigated using a nested PCR by targeting a highly conserved S/P region (332 bp) of the HBV genome as previously described [17, 18]. Amplification reactions were carried out in 25 µL (1x PCR buffer, 0.2 mM dNTPs, 0.4 µM specific primer, and 1U Taq DNA Polymerase (Qiagen GmbH, Hilden, Germany). Primers HBV-022, HBV-065, and HBV-066 were used for outer PCR, while primers HBV-024, HBV-041, and HBV-064 were used for inner PCR (see Additional file 1). The thermal cycling program for the outer PCR: initial denaturation at 95 °C for 15 min; followed by 35 cycles of denaturation (94 °C, 30 s), annealing (55 °C, 30 s), and extension (72 °C, 30 s); and a final extension of 5 min at 72 °C. For the inner PCR, the thermal parameters remained the same, except for the annealing step being at 54 °C. A positive control and a negative control were included in each run to validate the PCR results. The amplicons were visualized by agarose gel electrophoresis, and the positive samples were purified and cleaned using ExoSAP-IT PCR (Thermo Fisher Scientific, Waltham, MA, USA) and subsequently sequenced using the BigDye™ Terminator v.3.1 Cycle Sequencing Kit (Thermo Fisher Scientific, Waltham, MA, USA) on an Applied Biosystems 3130xl Genetic Analyzer (Applied Biosystems, Beverly, MA, USA).

### Quantitative real-time PCR

For the quantification of HBV DNA, real-time PCR was carried out using the SensiFAST™ one step RT-PCR kit (Meridian Biosciences, Memphis, Tennessee, USA) on a LightCycler 480 (Roche Diagnostics Corporation, Rotkreuz, Switzerland). Each RT-PCR reaction was performed in a volume of 20 µL, consisting of 0.8 µL each of 10 µM forward primer (HBV-61) and reverse primer (HBV-62), 10 µL RT-PCR Mix 2X, 0.3 µL Probe HBV TM-05 (see Additional file 1), 3.1 µL PCR grade water, and 5 µL of the template. The cycling condition was 5 min at 95 °C, followed by 45 cycles of 95 °C for 10 s and 60 °C for 34 s.

### Sequence analysis

The sequence was trimmed in Seqman version 6.1 (DNASTAR, Lasergene, USA) and the resulting consensus sequence was aligned using MAFFT version 7 (https://mafft.cbrc.jp/alignment/) with 17 references representative of genotypes A–H from NBCI genotyping tool (https://ncbi.nlm.nih.gov/projects/genotyping/) and 21 additional sequences obtained from BLAST results of the positive sequence. The alignment was done using the G-INS-I iterative refinement method. Phylogenetic analysis of the aligned sequences was performed using Mega 11 (https://megasoftware.net/). The phylogenetic tree was constructed using the neighbor-joining method. The tree was constructed using the model with the lowest BIC score and a bootstrap value of 1000. In this case, the K2 + G model was the best possible approximation. The tree was graphically adjusted using iTOL (https://itol.embl.de/). The sequence was examined for mutations using BioEdit version 7.2.6 and geno2pheno software (https://hbv.geno2pheno.org). The sequences were deposited to GenBank and was assigned the accession numbers: PQ441817 – PQ441825.

## Results

### Baseline characteristics

A total of 330 serum samples were collected from healthy blood donors at the Indonesian Red Cross in Jember. The donors were aged between 17 and 65 years, with 58 female donors and 272 male donors. All donors were from rural region and had a median age of 37 years, with an interquartile range (IQR) of 28 to 48 years. Male participants accounted for 82% of the samples (*n* = 272) and females for 18% (*n* = 58).

### HBV serology and nucleic acid testing

ELISA testing revealed that 27% (*n* = 89/330) tested positive for anti-HBs antibodies. This percentage was 15% (*n* = 49/330) among those who tested positive for anti-HBs antibodies but negative for anti-HBc antibodies, indicating immunity from vaccination. Notably, 6% of individuals tested positive for anti-HBc only, suggesting either resolved infections where anti-HBs levels have waned or potential OBI cases. In addition, 12% tested positive for both anti-HBc and anti-HBs, which typically indicates resolved infections (Table 1).

**Table 1:** Overall prevalence of anti-HBs and anti-HBc markers among the 330 tested blood serum samples

Serology	Percentage (*n*)
Anti-HBs positive and anti-HBc positive	12% (40/330)
Anti-HBs negative and anti-HBc positive	6% (21/330)
Anti-HBs positive and anti-HBc negative	15% (49/330)
Anti-HBs negative and anti-HBc negative	67% (220/330)

**Fig. 1:**
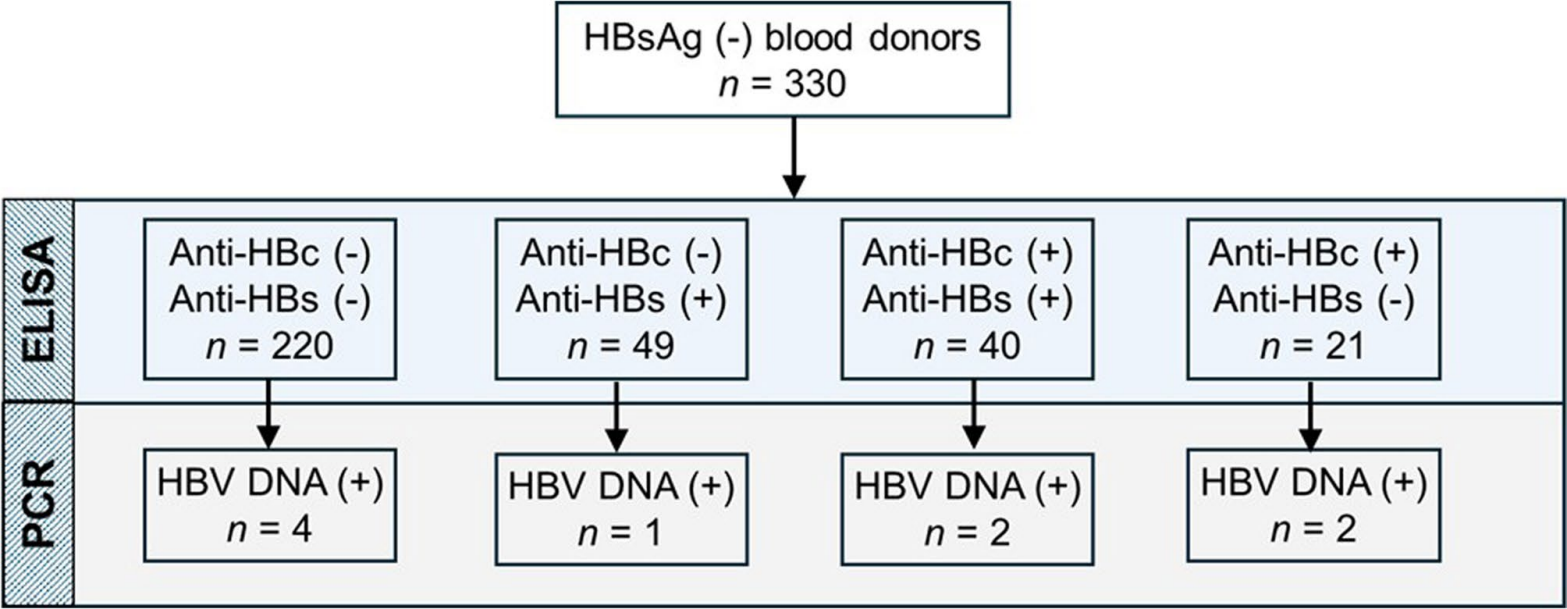
Study design and summary of results A total of 330 HBsAg (-) blood donors were screened for anti-HBc and anti-HBs by ELISA before nested PCR targeting a highly conserved S/P region of the HBV genome. Nine samples were found to be HBV DNA (+)

Nested PCR analysis detected HBV DNA in 3% (9/330) of the samples. All OBI-positive blood donors were male, aged between 26 and 59 years, with a median age of 41 years. Among them, four were seronegative for all hepatitis B antibodies, one was positive only for anti-HBs, two for anti-HBc, and two were seropositive for both anti-HBs and anti-HBc (Fig. 1). The three OBI cases with anti-HBs positivity had a mean anti-HBs level of 207.3 mIU/mL, lower than the overall study population mean of 225.2 mIU/mL. Similarly, the one OBI-positive individual with anti-HBs positivity alone had a low anti-HBs titer of 18 mIU/mL. Among the nine OBI positive cases, only one (sample #D59) had a quantifiable viral load of 275 IU/mL, while the remaining eight were below the detection limit by qPCR.

### OBI genotyping

**Fig. 2:**
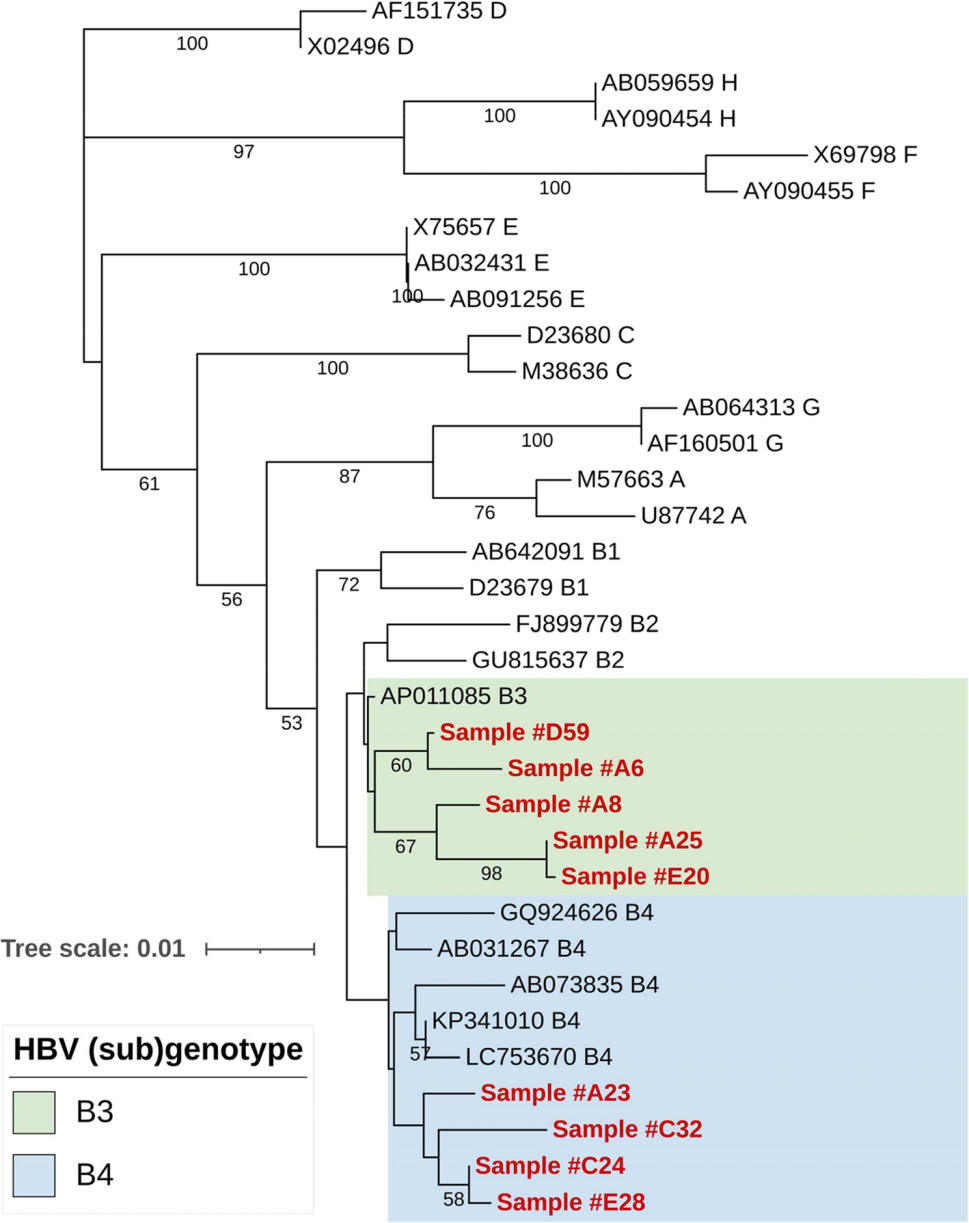
Phylogenetic tree of the S/P gene of HBV genome using the neighbour-joining method. The evolutionary distances were computed using Kimura 2-parameter (K2 + G) model with 25 reference nucleotide sequences representing genotypes A-H. The OBI sequences from this study clustered in the HBV subgenotypes B3 and B4. Bootstrap values larger than 50% are indicated. The OBI sequences are highlighted in red

**Fig. 3:**
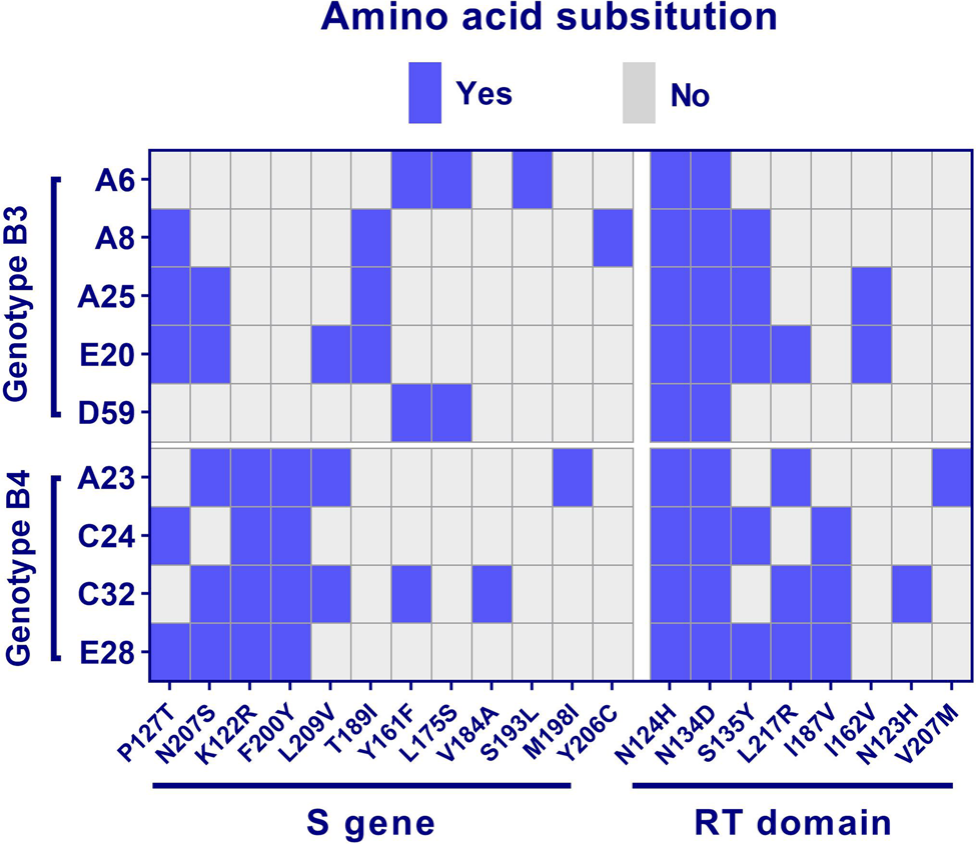
Distribution of mutations in the PreS/S gene and RT domain of Pol gene of OBI sequences

Sequencing of the PCR products revealed that all positive samples belonged to HBV genotype B, which is predominant in Indonesia as compared with available sequences in the NCBI database [6, 19, 20]. Phylogenetic analysis showed that the OBI-positive samples belonged to genotype B and were categorized into sub genotypes B3 and B4 (Fig. 2). For the genetic analysis, the most closely related references of sub genotypes B3 and B4 were used. A total of 12 amino acid substitutions in the surface (S) protein and another eight in the overlapping reverse transcriptase (RT) domain of the polymerase (Pol) protein were identified in our sequences collectively in comparison to the references. The number of mutations observed per sample ranged from two to six in the S protein and from two to five in the RT domain. In the S protein, the amino acid substitutions P127T and N207S were the most frequent, occurring in 55.6% of the samples (*n* = 5/9), followed by K122R and F200Y in 44.4% of the samples (*n* = 4/9). In the Pol protein, the N124H and N134D mutations were found in all positive samples (100%), followed by S135Y in 55.6% of samples (*n* = 5/9) and L217R in 44.4% of samples (*n* = 4/9). Other amino acid substitutions with lower frequencies are shown in Fig. 3.

## Discussion

In 2020, Vietnam had the highest prevalence of Hepatitis B in Southeast Asia (7.2%), followed by Indonesia with a rate of 6.4%. This rate is higher than those of other ASEAN member states, including Malaysia (2.3%), Thailand (2.56%), Cambodia (3%), and Lao PDR (3.5%) [21]. This highlights the critical need for measures to prevent the transmission of this viral infection in the region. Aligned with the World Health Organization’s Sustainable Development Goals, Indonesia has set a national target to eliminate the hepatitis epidemic by 2030 [22, 23]. Hepatitis B undoubtedly imposes a significant burden, both economically and in terms of disease progression, as it can develop into chronic HBV, cirrhosis, and increase mortality rates [2]. In 2013, the prevalence of hepatitis B virus in Indonesia was 7.1%, indicating a moderate to high burden of infection, and this prevalence remained at 6.4% by 2022 [24], contributing to a significant rate of OBI in the country. While numerous studies on OBI have been conducted in different regions of Indonesia, reporting prevalence rates ranging from 3.7% to 13%, there is a lack of data from specific areas such as Jember, underscoring the need for targeted OBI screening in these vulnerable populations in East-Java [6, 14, 15].

In this study, 330 HBsAg-negative samples were analyzed, revealing a positivity rate of 2.7% (*n* = 9/330) for occult HBV infection. This prevalence is notably higher than those reported in other Southeast Asian countries such as Hong Kong (1/4,255), Malaysia (1/13,602), Thailand (1/12,807), and Singapore (1/22,575) [25]. Also, among anti-HBc(+) donors in Taiwan, the prevalence was only 1/630 [25]. In Vietnam, a study reported an occult HBV infection positivity rate of 0.3% (*n* = 2/623), while an earlier study found a higher rate of 3.9% (*n* = 35/306) [11, 26]. In Malaysia, another study reported a 5.5% rate (*n* = 55/1000) among blood donors [27]. Variability in these findings may be attributed to factors such as differences in population demographics, HBV endemicity across geographic regions, criteria of blood donor selection, and variations in HBV genotypes.

Anti-HBs are neutralizing antibodies that indicate prior immunity to HBV and are usually the only detectable serological marker in vaccinated individuals [28]. In our cohort, 15% (*n* = 49/330) of blood donors were positive for anti-HBs alone, with a mean anti-HBs level of 225.2 mIU/ml, indicating a lower vaccination coverage than the national average. A low response is defined by anti-HBs levels between 10 and 99 mIU/ml, while a normal response is between 100 and 999 mIU/ml [29]. Excluding low and non-responders, only 10.9% (*n* = 36/330) of the cohort were normal responders, indicating a relatively small proportion with strong immunity. The seropositivity rate for anti-HBc in this cohort was 18% (*n* = 61/330), with 6% (*n* = 21/330) testing positive for anti-HBc only. This result may indicate resolved infections where anti-HBs levels have waned over time, or passive transmission of anti-HBc to infants born to HBsAg-positive mothers, or the presence of OBI [30]. Although OBI is more common in anti-HBc positive cases, cases of OBI with a seronegative profile have also been reported. Of the 21 anti-HBc positive samples considered primary suspected cases of OBI, two were confirmed as HBV DNA positive, indicating an OBI rate of 9.5% in anti-HBc positive individuals. In addition, seven samples from other serological groups tested positive by nucleic acid testing (NAT): two samples (5%) from the anti-HBc+/anti-HBs + group, indicating HBV DNA persistence despite serologically resolved infection; one sample (2%) from the anti-HBc–/anti-HBs + group, indicating immunity due to vaccination; and four samples (1.8%) from the seronegative group (anti-HBc–/anti-HBs–), indicating susceptibility to HBV infection in these individuals.

The positive samples all belonged to HBV – B genotype. While genotype B is associated with high HBsAg clearance and earlier HBeAg seroconversion, which contributes to a better prognosis, it has also been associated with the integration of HBV DNA into the host genome, a key factor in the development of HCC. Notably, genotype B has been associated with an earlier onset of HCC compared to other genotypes, especially in younger individuals. Although genotype B generally responds better to interferon-based therapy and is less likely to develop cirrhosis, the increased risk of HCC due to HBV integration warrants careful monitoring, especially in the case of occult HBV infection [31].

Several host and viral factors have been associated with the pathogenesis of OBI, including mutations in the S gene, but studies on genetic markers are inconclusive and in vivo studies are lacking [32]. Multiple amino acid substitutions identified in this study were well-characterized as escape mutants influencing HBsAg secretion. For instance, the frequently observed P127T amino acid substitution in five OBI-positive cases, was shown to alter the conformation of the ‘a’ determinant region of the S protein, impacting its antigenic properties and immune recognition [33]. This locus is under selective pressure, likely driven by immune selection following Hepatitis B vaccination, enabling the virus to evade immune detection and persist as OBI [30]. In addition, another common mutation, K122R, which occurs in 44% of samples, is associated with immune escape and diagnostic failure, particularly because of its role as a serotype-determining amino acid [34]. However, the specific role of this amino acid change in escape properties requires further investigation. In addition, the Y161F mutation, located in the major hydrophilic region of HBsAg, is observed in 3 samples, and has previously been linked specifically to OBI cases, suggesting a role in immune evasion [35]. The Y206C mutation, detected in one of samples, has been linked to reduced viral replication, lower HBsAg levels, and less ALT activity, suggesting a less severe infection profile [36]. In the analysis of the RT domain of the Pol gene from positive samples, several notable mutations were identified. The N124H and N134D mutation was present in 100% of OBI samples, although its significance remains unclear. The S135Y mutation, detected in 56% of samples, has been commonly documented in treatment-naïve patients with chronic hepatitis B, particularly in genotype D. This mutation is also implicated in the failure of lamivudine treatment [37]. Another significant mutation, L217R, found in 44% of samples, is associated with adefovir resistance, particularly in patients with HBV subgenotype A2. And the I187V and N123H mutations, found in three and one OBI samples respectively, are both rare and associated with entecavir resistance. The V207M mutation, found in one sample, is often combined with other mutations contributing to lamivudine resistance [38]– [39]. These findings highlight the diversity of mutations in the PreS/S region and RT domain in OBI patients and their potential impact on diagnosis and antiviral treatment outcomes.

In many countries, anti-HBc positivity is followed by nucleic acid testing to identify occult hepatitis B infection among HBsAg-negative individuals. However, in Indonesia, screening relies solely on HBsAg detection, which significantly increases the risk of undetected OBI transmission and reactivation, particularly in high-risk populations. This study marks the first investigation into OBI among blood donors in Jember, Indonesia, revealing a low prevalence rate among HBsAg-negative individuals despite the high HBV burden in the country. In addition, several key mutations such as P127T and S135Y were observed in association with immune escape and drug resistance, emphasizing the complexity of OBI in this population. Our findings highlight the high prevalence of OBI in anti-HBc-positive individuals, supporting the use of NAT in routine blood donor screening. We recommend that the Indonesian government incorporate NAT, especially for anti-HBc-positive donors, and/or adopt pooled NAT strategies to further reduce HBV transmission risks.

Taken together, this study offers valuable insights into the prevalence and mutational characteristics of OBI among blood donors in Jember but has several limitations. Firstly, total anti-HBc testing was used instead of distinguishing IgG from IgM anti-HBc, which could better differentiate between past and recent infections. Secondly, we lacked data on the sensitivity of HBsAg testing at the Indonesian Red Cross, potentially affecting OBI detection rates. Future research should refine diagnostic methods, evaluate HBsAg assay sensitivity across different settings, and include broader demographic and regional analyses. Longitudinal studies on HBV markers could enhance understanding of OBI persistence and its clinical implications. Expanding screening, particularly with NAT for high-risk donor groups, will improve blood safety and public health measures against HBV transmission.

## Supplementary Information

Supplementary Material 1.

## Data Availability

All data obtained or analysed in this study are included in this article. A total of 9 successfully sequenced samples with accession numbers PQ441817 to PQ441825 were submitted to the NCBI GenBank database.
