# Supplementary material for: Prevalence of occult hepatitis B infection and hepatitis B genotype characterization among blood donors in Jember Regency, East Java, Indonesia
Source: BMC Infect Dis. 2025 Oct 31;25:1462. doi: 10.1186/s12879-025-11864-9 (PMC12577327; doi:10.1186/s12879-025-11864-9)
Supplement: Supplementary file 1 — Supplementary Material 1. [file 12879_2025_11864_MOESM1_ESM.docx]

**Supplementary Materials**

**Additional file 1.** Primer and probe sequences used in the study.

| **Primer** | **Sequence (5´-3´)** |
| --- | --- |
| HBV-022 | TGCTGCTATGCCTCATCTTC |
| HBV-066 | CACAGATAACAAAAAATTGG |
| HBV-065 | CAAAGACAAAAGAAAATTGG |
| HBV-024 | CAAGGTATGTTGCCCGTTTGTCCT |
| HBV-041 | GGACTCAMGATGYTGCACAG |
| HBV-064 | GGACTCACGATGCTGTACAG |
| HBV-61 | GGACCCCTGCTCGTGTTACA |
| HBV-62 | GAGAGAAGTCCACCACGAGTCTAGA |
| HBV-TM-5 | FAM5'-TGTTGACAARAATCCTCACAATACCRCAGA-3' DabCyl |
